# Preclinical Assessment of a New Polyvalent Antivenom (Inoserp Europe) against Several Species of the Subfamily Viperinae

**DOI:** 10.3390/toxins11030149

**Published:** 2019-03-05

**Authors:** Alejandro García-Arredondo, Michel Martínez, Arlene Calderón, Asunción Saldívar, Raúl Soria

**Affiliations:** 1 Laboratorio de Investigación Química y Farmacológica de Productos Naturales, Facultad de Química, Universidad Autónoma de Querétaro, Querétaro 76010, Mexico; 2 Veteria Labs, S.A. de C.V. Lucerna 7, Col. Juárez, Del. Cuauhtémoc, Ciudad de México 06600, Mexico; mmartinez@veterialabs.com (M.M.); asaldivar@veterialabs.com (A.S.); 3 Inosan Biopharma, S.A. Arbea Campus Empresarial, Edificio 2, Planta 2, Carretera Fuencarral a Alcobendas, Km 3.8, 28108 Madrid, Spain; arlene.calderon@veterialabs.com (A.C.); rsoria@inosanbiopharma.com (R.S.)

**Keywords:** *Viperinae*, *Vipera*, *Macrovipera*, *Montivipera*, neutralization, paraspecificity, antivenom

## Abstract

The European continent is inhabited by medically important venomous Viperinae snakes. *Vipera ammodytes*, *Vipera berus*, and *Vipera aspis* cause the greatest public health problems in Europe, but there are other equally significant snakes in specific regions of the continent. Immunotherapy is indicated for patients with systemic envenoming, of which there are approximately 4000 annual cases in Europe, and was suggested as an indication for young children and pregnant women, even if they do not have systemic symptoms. In the present study, the safety and venom-neutralizing efficacy of Inoserp Europe—a new F(ab’)_2_ polyvalent antivenom, designed to treat envenoming by snakes in the Eurasian region—were evaluated. In accordance with World Health Organization recommendations, several quality control parameters were applied to evaluate the safety of this antivenom. The venom-neutralizing efficacy of the antivenom was evaluated in mice and the results showed it had appropriate neutralizing potency against the venoms of several species of *Vipera*, *Montivipera*, and *Macrovipera*. Paraspecificity of the antivenom was demonstrated as well, since it neutralized venoms of species not included in the immunization schemes and contains satisfactory levels of total proteins and F(ab’)_2_ fragment concentration. Therefore, this new polyvalent antivenom could be effective in the treatment of snake envenoming in Europe, including Western Russia and Turkey.

## 1. Introduction

The European continent is inhabited by medically important venomous snakes of the subfamily Viperinae [1,2]. According to the World Health Organization, the venomous snakes causing the greatest public health problems in Europe are *Vipera ammodytes*, *Vipera berus*, and *Vipera aspis* [2]. *V. berus*, usually known as European adder, is extremely widespread in Europe [1,3]. *V. ammodytes*, commonly known as nose-horned viper, is considered the most venomous European snake and primarily inhabits the southern and eastern regions of Europe [1,3,4]. The distribution of *V. aspis* is limited to Western Europe, including France, Switzerland, and Italy [1,3]. In addition, there are other equally significant snakes in some specific regions, *Montivipera xanthina* and *Macrovipera lebetina* are the most dangerous species in Turkey [5]; *M. xanthina* is also distributed around Central Europe and the Middle East, whereas *Macrovipera lebetina* is distributed in Eastern Europe, the Middle East, Northern Africa, Central Asia, and South Asia [1].

In an epidemiological study, the reported average number of snakebites in Europe—including Western Russia and Turkey—was around 7500 cases per year (1.06 per 100,000 inhabitants), with more than 90% of victims being hospitalized and 0.05% died [3]. 

In general, the clinical symptoms caused by snakebites in Europe are classified into local or systemic. Local symptoms may include pain, swelling, redness, edema, ecchymosis, necrosis, and numbness. The systemic symptoms include tachycardia, hypotension, anaphylaxis, nausea, vomiting, abdominal pain, hemorrhagic syndrome, pulmonary bleeding, coagulopathy, and neurotoxicity [4,6,7,8].

A clinical gradation of the envenoming caused by viper bites classifies the symptoms into the following: grade 0, fang marks and absence of local and symptoms; grade 1, local edema and absence of systemic symptoms; grade 2, regional edema and moderate systemic symptoms; and grade 3, extensive edema and severe systemic symptoms [9,10]. In a posterior classification, grade 2 was divided into 2a (regional edema and/or hematoma) and 2b (grade 2a symptoms associated with systemic signs or biological abnormalities) [11].

Mortality due to snakebite in Europe is not a major problem, compared to Africa and India, but cases of snakebite have usually required hospitalization and an appropriate treatment. At the present time, use of highly purified immunoglobulin fragments dramatically reduce both the severity and mortality of snakebites [3]. Immunotherapy is indicated for systemic envenoming (grades 2 and 3), and has been recommended for young children, pregnant women, and patients with progressive swelling, even if they are not classified as grade 2 or 3 [3,4].

For regions inhabited by several medically important snake species, the World Health Organization recommends the manufacture of polyspecific antivenoms that are effective against all possible regional snake varieties [2]. This is important because, as a rule, patients cannot identify the species that bit them; thus, an expert analysis of the shape of the snakebite is required [12]. In the present study, the safety and venom-neutralizing efficacy of Inoserp Europe—a new F(ab’)_2_ polyvalent antivenom, designed to cover envenoming caused by medically important snakes of the Eurasian region—were evaluated.

## 2. Results

### 2.1. Physicochemical and Biochemical Characteristics of the Antivenom

Lyophilized Inoserp Europe antivenom is a sterile lyophilized white powder formulated to be reconstituted with 10 mL of sterile water for injection. The reconstitution time of the lyophilized powder was 22 s, producing a colorless to pale yellow transparent solution with a total protein concentration of 17.4 mg/mL, and a final pH value of 6.81 at 25.3 °C.

**Figure 1:**
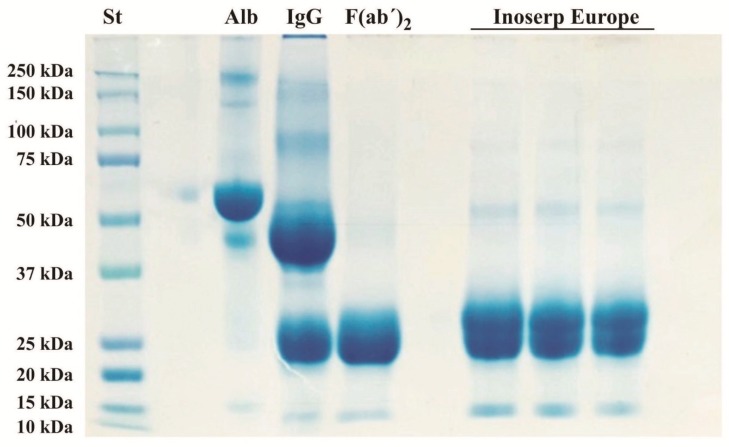
Qualitative analysis of the antivenom by SDS-PAGE under reducing conditions (12% acrylamide gels). The protein profile of the antivenom (25 µg of protein) was compared with 10 µL of a Precision Plus Protein Kaleidoscope standard (St), 15 µg of equine serum albumin (alb), 15 µg of purified horse IgG (IgG), and 15 µg of purified horse IgG F(ab’)_2_ [F(ab’)_2_]. Protein bands were visualized with a Coomassie premixed staining solution.

Qualitative analysis of the antivenom by SDS-PAGE under reducing conditions revealed two prominent bands of approximately 25 kDa (Figure 1), corresponding to the digested heavy and light chains of reduced F(ab’)_2_ fragments. 

**Figure 2:**
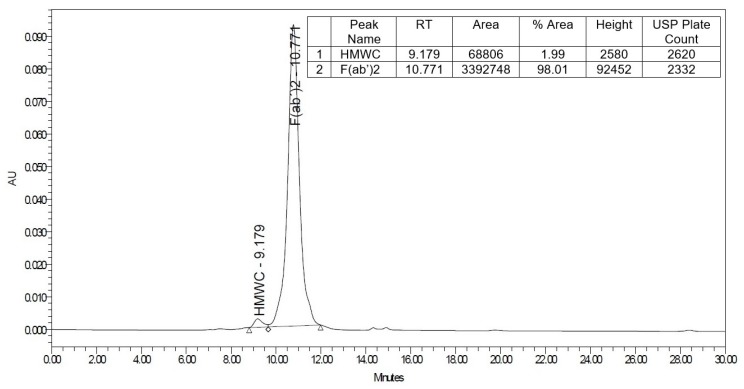
Quantitative analysis of the antivenom by size-exclusion chromatography.

In addition, quantitative analysis of the antivenom by size-exclusion chromatography showed that F(ab’)_2_ fragments comprise 98.01% of its total composition (Figure 2).

### 2.2. Neutralization of Lethality (Paraspecificity Evaluation)

Table 1 shows results of the determination of lethal activity of the venoms used in this study to determine the paraspecificity of Inoserp Europe antivenom (Figure S1). The venoms of *Vipera berus berus*, *Montivipera raddei raddei* (both from Turkey), and *Vipera latifii* from Iran showed slightly higher lethal activity than the others. The venoms of the genus *Macrovipera* showed lower lethal activity than that of the other venoms. The other venoms presented median lethal dose values (LD_50_) in a range from 7.03 to 12.78.

**Figure 3:**
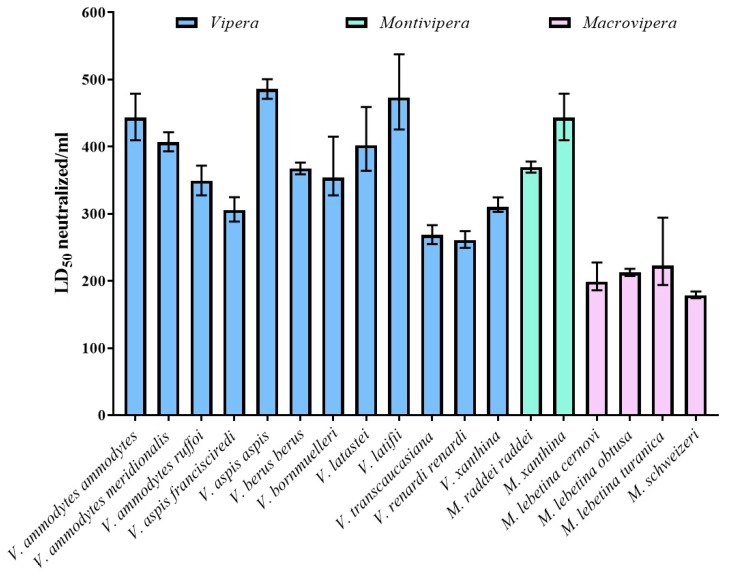
Results of the paraspecificity of the antivenom.

Table 2 and Figure 3 summarize the results of the paraspecificity of the antivenom. Smaller amounts of antivenom were required to neutralize the lethal activity of the venoms of the species used in its production. Nevertheless, Inoserp Europe antivenom effectively neutralized five times the LD_50_ of all the venoms analyzed in this study, which demonstrates its cross-neutralization and paraspecific neutralization.

## 3. Discussion

In Europe, the epidemiological studies of snakebites show that mortality is not a serious problem; nevertheless, most cases required hospitalization and a rapid recognition of symptoms because it is essential to decide if immunotherapy is required [3,4,6,12,13]. Usually, immunotherapy with antivenoms is indicated only to patients with systemic envenoming (approximately 4,000 annual cases in Europe), but it was suggested that antivenom should be indicated to young children and pregnant women—representing 50% of snakebite cases—even if they do not have systemic symptoms [3,4]. In a previously reported descriptive review, eight antivenoms available for the treatment of European *Vipera* spp. envenoming were identified, and the need of more preclinical data to ensure the efficacy of those antivenoms was mentioned [14]. In fact, the World Health Organization strongly suggests the assessment of several quality control parameters and preclinical neutralization in order to guarantee the safety and efficacy of the antivenoms [2]. 

In the present study, the safety and venom-neutralizing efficacy of a new lyophilized and non-pyrogenic polyvalent F(ab’)_2_ antivenom against the venoms of several medically important venomous snakes of Europe were assessed. The chosen active substances of this antivenom are F(ab’)_2_ fragments because they offer advantages over IgG, which is associated with undesirable effects [3]. Moreover, some authors suggest that F(ab’)_2_ antivenoms are more effective than Fab antivenoms because they have a longer half-life in the serum compartment and no additional doses are needed [15,16]. 

The assessment of some biochemical and physicochemical characteristics of the antivenoms is necessary to ensure their safety. For example, high levels of albumin or other contaminants, like Fc fragments in the antivenoms or antivenoms with a high concentration of total protein, have been associated with early adverse reactions in patients treated with immunotherapy [2,17]. For these reasons, the World Health Organization recommends that the total protein concentration of the antivenoms should not exceed 10 g/dL, and they also recommend that immunoglobulins or their fragments should constitute more than 90% of that value [2]. The antivenom produced in the present study meets those quality requirements: it contains a total protein concentration of 17.4 mg/mL (1.74 g/dL), of which 98.01% are F(ab’)_2_ fragments. This antivenom contains a lower quantity of protein per dose than the antivenoms available for the treatment of European *Vipera* spp. envenoming [14], and the analysis by electrophoresis and chromatography show that the major constituents of this antivenom are F(ab’)_2_ immunoglobulin fragments. These data show that Inoserp Europe antivenom has advantages over snake antivenoms that are currently used in humans in Europe. However, it is important to consider that, like other antivenoms, Inoserp Europe may contain some antibody fragments that are not related to the venoms.

Inoserp Europe is a polyvalent antivenom designed to cover envenoming caused by medically important snakes of Europe, including Western Russia and Turkey. The species causing the greatest public health concern in Europe are *Vipera ammodytes*, *V. berus*, and *V. aspis* [1,2]. The nose-horned viper, *V. ammodytes*, is considered the most venomous European snake and is mainly distributed in Central Asia, the Middle East, Central Europe, and Western Europe [1,3,4]. The venom of this species induces local effects like extensive swelling, edema, and ecchymosis [7]. This venom induces important hemostatic alterations that results in systemic effects like hemorrhagic syndrome, pulmonary bleeding, and coagulopathy [4]. In addition, this venom also induces cardiotoxicity and neurotoxicity, which in some cases results in vessel and myocardial dysfunction and cranial nerve paresis or paralysis [7,18,19]. 

The European adder, *V. berus*, is the medically important venomous snake most widely distributed in Europe, and is widely found in Eastern Europe, Western Europe, Central Europe, Central Asia, and East Asia [1,2]. Its main venom symptoms are hemorrhagic effects [20], and the systemic symptoms include dizziness, tachycardia, hypotension, shock, and gastrointestinal symptoms [21,22,23]. Nevertheless, it has been reported that the venom of *V. berus* also induces neurotoxic activity [24,25], which is due to individual intra-population variability in the venom composition [26].

*V. aspis* is the other significant European venomous snake distributed in Western Europe; this includes France, Switzerland, and Italy [1,3]. Its symptoms are similar to those provoked by the venom of *V. berus* and also include neurotoxic effects [20]. There are other medically important snakes in some specific regions of Europe [1,2]. For example, *Macrovipera lebetina* is distributed in Eastern Europe, the Middle East, Northern Africa, and Central and South Asia [1], and is one of the most dangerous species in Turkey [5]. *Montivipera xanthina* is another of the most dangerous species in Turkey, and is also distributed around central Europe and the Middle East [1,5]. 

Snake venoms are highly complex: they are biologically active mixtures that typically contain several enzymatic and non-enzymatic components. Most of these are peptides and proteins with neurotoxic, hemolytic, proteolytic, or cytotoxic properties [23,27]. Studies of the proteome and peptidome of *Vipera* venoms suggest broad similarities in their composition, but it is clear that there are variations in the toxin composition among species and subspecies that determine the differences in their mechanisms of envenoming [7,20,26,27,28,29]. Therefore, the use of polyvalent antivenoms increases the efficacy of the treatment.

In order to assess the venom-neutralizing efficacy of Inoserp Europe antivenom in this study, the lethality of the venoms in mice was first determined. As expected, the venoms of genera *Vipera* and *Montivipera* species were the most lethal. In general, the venoms analyzed in this study had LD_50_ valuesin a similar range to those previously reported in other studies [21,30,31]. The results of the preclinical neutralization showed that Inoserp Europe antivenom effectively neutralized the lethality of all venoms analyzed in this study, demonstrating its paraspecificity, since it neutralized venoms of species not included in the immunization schemes, like *Montivipera raddei raddei*, *V. xanthina*, *V. renardi renardi*, *V. transcaucasiana*, *V. latifii*, and *V. bornmuelleri*. The World Health Organization classified some of these snake species as being of secondary medical importance (category 2), since they are highly venomous but less frequently involved in clinical cases [1,2]. It is noteworthy to mention that when comparing the potencies of this antivenom with those of other products, Inoserp Europe antivenom seems to be more effective [30,31]. These data are important because they clearly suggest that Inoserp Europe antivenom could be highly effective for the treatment of envenoming caused by diverse European vipers of the genera *Vipera*, *Montivipera*, and *Macrovipera*, including the species causing the greatest public health in the Eurasian region. This study has some limitations. The venoms of *V. ammodytes*, *V. berus*, and *V. aspis* cause coagulopathy and neurotoxicity in humans [4,7,18,19,20,21,22,23,24,25], and so the ability of Inoserp Europe antivenom to neutralize the hemorrhagic, procoagulant, necrotizing, and neurotoxic activities of these venoms needs further study.

## 4. Conclusions

In summary, this study provides evidence of the safety and venom-neutralizing efficacy of Inoserp Europe, a new polyvalent equine F(ab’)_2_ antivenom. This antivenom contains satisfactory levels of total proteins (1.74 g/dL) and F(ab’)_2_ fragment concentration (98.01%); the antivenom also has appropriate neutralizing potency against the venoms of several species of the genera *Vipera*, *Montivipera*, and *Macrovipera*. The paraspecificity of the antivenom was demonstrated by its ability to neutralize venoms of species not included in the immunization schemes. This antivenom could be effective in the treatment of snake envenoming in Europe, including western Russia and Turkey.

## 5. Materials and Methods 

### 5.1. Venoms

The venoms used in the present study were obtained as certified lyophilized powders from Latoxan, S.A.S. (France), and Alphabiotoxine Laboratory (Belgium).

### 5.2. Animal Handling

Hyperimmune plasma production was developed in the Vivarium of Veteria Labs (Puebla, Mexico). In order to determine the neutralizing potency of the antivenom, CD1 mice of either sex weighing 18 to 20 g were used. The animals were supplied by Unidad de Producción y Experimentación de Animales de Laboratorio (UPEAL) from Centro de Investigación y de Estudios Avanzados del Instituto Politécnico Nacional (CINVESTAV). The animals were maintained with free access to standard mouse food pellets and water ad libitum. All experiments were carried out following the Official Standard NOM-062-ZOO-1999 for the production, care, and use of laboratory animals. This study was based on the guidelines and approvals of the Preclinical Research Unit Ethics Committee (UNIPREC); code: BPL-002/15; the date of the approval: 16 June 2016.

### 5.3. Antivenom Production

Inoserp Europe antivenom is a lyophilized preparation of equine F(ab’)_2_ immunoglobulin fragments produced according to the protocols recommended by WHO [2]. Horse hyperimmune plasmas were produced by immunization with a mixture of lyophilized pools of venoms of the following European species: *Vipera ammodytes, Vipera aspis, Vipera berus, Vipera latastei, Montivipera xanthina, Macrovipera schweizeri, Macrovipera lebetina obtuse, Macrovipera lebetina cernovi*, and *Macrovipera lebetina turanica*. Hyperimmune plasmas were collected from whole blood with anticoagulant. 

F(ab’)_2_ fragments were obtained by enzymatic digestion with pepsin and precipitation with ammonium sulfate. Fc and other components from the enzymatic digestion were removed by filtration. After further filtration, concentration and washing, tangential flow filtration was performed on the crude total F(ab’)_2_. Finally, the product was sterilized and concentrated to yield the formulated bulk product, then it was lyophilized. The protein content was determined using the Bradford assay [32] and a standard curve prepared with lyophilized IgG.

### 5.4. Sodium Dodecyl Sulfate-Polyacrylamide Gel Electrophoresis (SDS-PAGE)

Electrophoretic analyses of the antivenom were performed as previously described by Laemmli [33] under reducing conditions. The samples were diluted 1:1 in a sample buffer buffer (Cat # 161-0737, Bio-Rad, Hercules, CA, USA) containing β-mercaptoethanol, and were then heated at 95 °C for 5 min. Then, the samples were loaded in 12% polyacrylamide gels and were electrophoresed at 80 V for 1 h and then at 100 V for 45 min, using Tris-glycine-SDS as buffer (25 mM Tris, 192 mM glycine, pH 8.3, Bio-Rad, Cat # 161-0734). Protein bands were visualized using Coomassie stain G-250 (Bio-Rad, Cat # 161-0786). Molecular masses were determined by comparison with Precision Plus Protein Kaleidoscope (Bio-Rad, Cat # 161-0375). The antivenom profile (25 μg of protein) was compared with the profiles of 15 μg equine serum albumin (Cat # ESA-BSH, RMBIO, Missoula, MT, USA), 15 μg purified horse IgG (Fitzgerald, Cat # 31R-1055, (Cat # 31R-1055, Fitzgerald, North Acton, MA, USA), and 15 μg purified horse F(ab’)_2_ (Fitzgerald, Cat # 31C-CH0807).

### 5.5. Analysis by High-Performance Liquid Chromatography (HPLC)

An analysis of the antivenom was carried out by size-exclusion chromatography in an HPLC system (Waters 1515, Milford, MA, USA) coupled with UV detector (Waters 2489). Samples of 20 μL of antivenom (1.65 mg/mL) were submitted to a BioSuite 250 SEC analytical column (10 μm, 10 × 300 mm, Waters) eluted with 0.1 M phosphate buffer, pH 6.7, at a flow rate of 1.7 mL/min. The absorbance was read at 280 nm.

### 5.6. Neutralization of Lethality (Paraspecificity Evaluation)

The median lethal dose (LD_50_) of each venom was determined according to the protocols recommended by WHO [2]. In other words, different doses of venom, dissolved in sterile saline solution (0.15 M NaCl) at a final volume of 0.5 mL, were injected intravenously in groups of five mice. The deaths occurring within 48 h were recorded, and the LD_50_ value of each venom was calculated by fitting a log dose–response curve using nonlinear regression analysis. Venom LD_50_ is defined as the minimal amount of venom causing death in 50% of the injected mice, and in this study, was expressed in μg venom/mouse.

In order to determine the neutralization potency of the antivenom, different doses of antivenom were pre-incubated with each venom (5 × LD_50_) and dissolved in sterile saline solution for 30 min at 37 °C [2]. After incubation, volumes of 500 μL of the mixture were injected intravenously in groups of five mice. The period of observation was 48 h, and the percentage of survival was used to calculate the median effective dose (ED_50_). The ED_50_ value is defined as the quantity of antivenom that protects 50% of injected mice, and was expressed in μL antivenom that neutralizes 5 × LD_50_, mg venom neutralized by mL antivenom, mg venom neutralized by vial of antivenom, and number of LD_50_ of venom neutralized by vial of antivenom.

### 5.7. Data Analysis and Statistics

Data and statistical analyses were performed in Prism 7.0 (GraphPad Software, Inc., San Diego, CA, USA). The LD_50_ and ED_50_ values were interpolated by fitting log dose–response curves using nonlinear regression analysis and all results were expressed as means and 95% confidence limits. Values were considered significantly different if there was no overlap of the 95% confidence limits.

## Figures and Tables

**Table 1:** Geographic origin and median lethal dose values (LD_50_) of the venoms used in the present study determined by intravenous injection in mice (n = 5). LD_50_ are expressed in µg of venom/mouse (18–20 g). 95% confidence intervals are included in parentheses.

Venom	Origin	LD_50_ in µg/Mouse (95% CI)
Vipera ammodytes ammodytes ^**^	Albania	8.07 (7.48–8.54)
Vipera ammodytes meridionalis ^**^	Greece	7.34 (6.20–8.16)
Vipera ammodytes ruffoi ^**^	Italy	8.29 (7.14–8.95)
Vipera aspis francisciredi ^**^	Switzerland	12.78 (10.51–14.05)
Vipera aspis aspis ^*^	France	8.42 (7.65–9.38)
Vipera berus berus ^*^	Turkey	5.28 (5.06–5.48)
Vipera bornmuelleri ^*^	Lebanon	11.32 (11.14–11.52)
Vipera latastei ^*^	Spain	8.17 (7.09–9.01)
Vipera latifii ^*^	Iran	5.52 (4.88–6.16)
Vipera transcaucasiana ^**^	Turkey	8.13 (6.94–9.03)
Vipera renardi renardi ^**^	Romania	11.84 (10.91–12.70)
Vipera xanthina ^*^	Turkey	7.03 (6.85–7.16)
Montivipera raddei raddei ^**^	Turkey	4.08 (3.21–4.59)
Montivipera xanthina ^**^	Turkey	7.17 (5.87–8.10)
Macrovipera lebetina cernovi ^*^	Turkmenistan	19.71 (18.34–20.60)
Macrovipera lebetina obtusa ^*^	Azerbaijan	16.32 (15.73–16.93)
Macrovipera lebetina turanica ^*^	Russia	18.36 (17.17–19.30)
Macrovipera schweizeri ^**^	Greece	17.32 (16.87–18.11)

* Obtained from Latoxan, S.A.S.; ** Obtained from Alphabiotoxine Laboratory.

**Table 2:** Median effective dose values (ED_50_) of the antivenom against the venoms tested expressed in µL of antivenom that neutralize 5 × LD_50_, mg of venom neutralized by ml of antivenom, mg of venom neutralized by vial of antivenom, and number of LD_50_ of venom neutralized by vial of antivenom. 95% confidence intervals are included in parentheses.

Venom	ED_50_ in µL (95% c.i.)	ED_50_ in mg/mL (95% CI)	ED_50_ in mg/vial (95% CI)	LD_50_ Neutralized/vial (95% CI)
*Vipera ammodytes ammodytes*	11.28 (10.44–12.21)	3.58 (3.30–3.86)	35.77 (33.05–38.65)	4432.6 (4095.0–4789.3)
*Vipera ammodytes meridionalis*	12.29 (11.86–12.72)	2.99 (2.89–3.09)	29.86 (28.85–30.94)	4068.3 (3930.8–4215.9)
*Vipera ammodytes ruffoi*	14.32 (13.45–15.26)	2.89 (2.72–3.08)	28.95 (27.16–30.82)	3491.6 (3276.5–3717.5)
*Vipera aspis francisciredi*	16.35 (15.39–17.33)	3.91 (3.69–4.15)	39.08 (36.87–41.52)	3058.1 (2885.2–3248.9)
*Vipera aspis aspis*	10.29 (9.99–10.61)	4.09 (3.97–4.22)	40.94 (39.70–42.18)	4859.1 (4712.5–5006.0)
*Vipera berus berus*	13.61 (13.29–13.94)	1.94 (1.89–1.98)	19.38 (18.92–19.85)	3673.8 (3586.8–3762.2)
*Vipera bornmuelleri*	14.14 (12.05–15.26)	4.00 (3.71–4.70)	40.03 (37.09–46.97)	3536.1 (3276.5–4149.4)
*Vipera latastei*	12.44 (10.89–13.73)	3.28 (2.98–3.75)	32.84 (29.75–37.51)	4019.3 (3641.7–4591.4)
*Vipera latifii*	10.57 (9.30–11.75)	2.61 (2.35–2.97)	26.12 (23.50–29.69)	4730.4 (4255.3–5376.9)
*Vipera transcaucasiana*	18.63 (17.66–19.65)	2.18 (2.07–2.30)	21.82 (20.73–23.02)	2683.8 (2549.7–2831.3)
*Vipera renardi renardi*	19.15 (18.23–20.06)	3.09 (2.95–3.25)	30.91 (29.51–32.47)	2611.0 (2492.5–2742.7)
*Vipera xanthina*	16.13 (15.41–16.51)	2.18 (2.13–2.28)	21.78 (21.28–22.80)	3099.8 (3028.5–3244.5)
*Montivipera raddei raddei*	13.53 (13.23–13.84)	1.51 (1.47–1.54)	15.08 (14.74–15.42)	3695.5 (3612.7–3779.3)
*Montivipera xanthina*	11.28 (10.44–12.21)	3.18 (2.94–3.43)	31.78 (29.36–34.34)	4432.6 (4095.0–4789.3)
*Macrovipera lebetina cernovi*	25.14 (21.99–26.87)	3.92 (3.67–4.48)	39.20 (36.68–44.82)	1988.9 (1860.8–2273.8)
*Macrovipera lebetina obtusa*	23.53 (22.94–24.11)	3.47 (3.38–3.56)	34.68 (33.84–35.57)	2124.9 (2073.8–2179.6)
*Macrovipera lebetina turanica*	22.42 (16.99–25.78)	4.09 (3.56–5.40)	40.95 (35.61–54.03)	2230.2 (1939.5–2942.9)
*Macrovipera schweizeri*	27.96 (27.13–28.66)	3.10 (3.02–3.19)	30.97 (30.22–31.92)	1788.3 (1744.6–1843.0)

## Supplementary Materials

The following are available online at https://www.mdpi.com/2072-6651/11/3/149/s1, Figure S1: Comparison of the median lethal dose values (LD50) of the venoms used in the present study determined by intravenous injection in in mice.
