# Supplementary material for: Preclinical Assessment of a New Polyvalent Antivenom (Inoserp Europe) against Several Species of the Subfamily Viperinae
Source: Toxins (Basel). 2019 Mar 5;11(3):149. doi: 10.3390/toxins11030149 (PMC6468668; doi:10.3390/toxins11030149)
Supplement: Supplementary file 1 [file toxins-11-00149-s001.pdf]

Supplementary Materials: Preclinical Assessment of A New Polyvalent Antivenom (Inoserp Europe) against Several Species of the Subfamily Viperinae

Alejandro García-Arredondo, Michel Martínez, Arlene Calderón, Asunción Saldívar and Raúl Soria

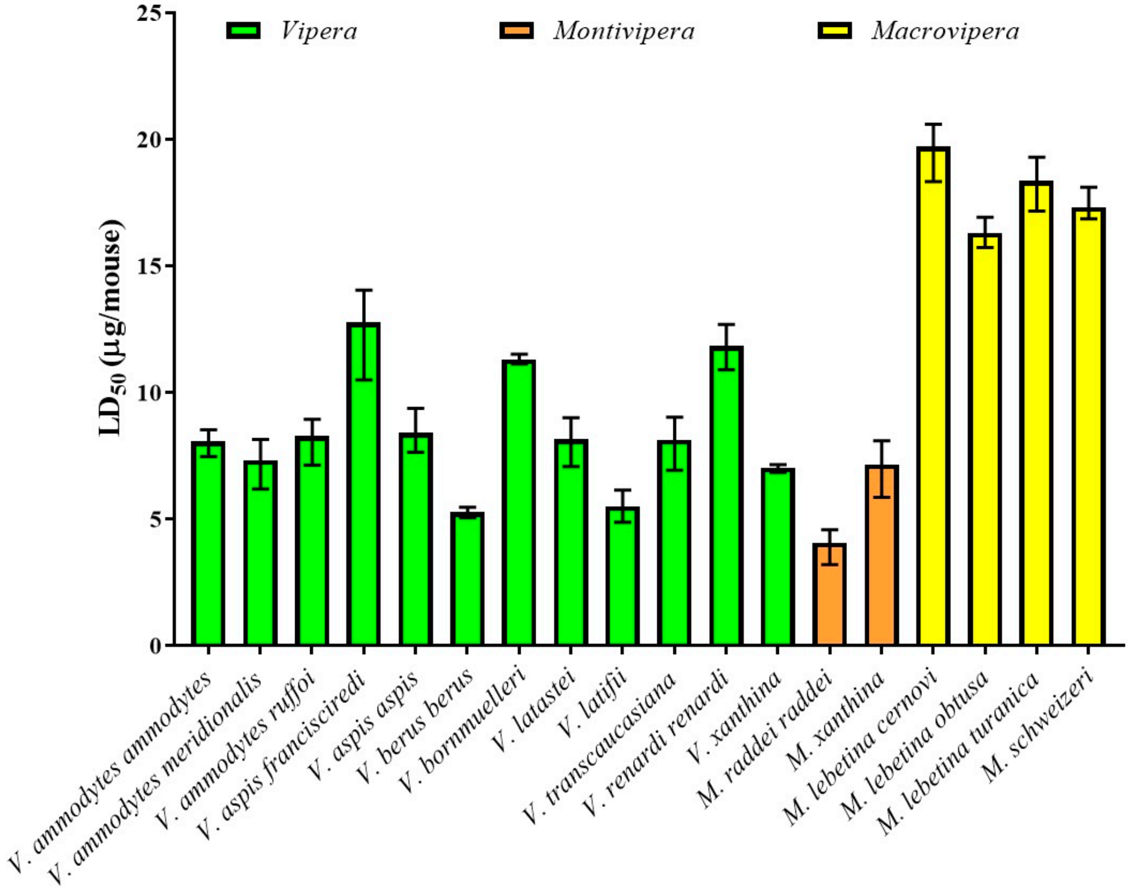

Figure S1. Comparison of the median lethal dose values (LD_{50}) of the venoms used in the present study determined by intravenous injection in mice. LD_{50} are expressed in µg of venom/mouse (18–20 g). 95% confidence limits are included as error bars.
